# Potential Biomarkers Associated with Multiple Sclerosis Pathology

**DOI:** 10.3390/ijms221910323

**Published:** 2021-09-25

**Authors:** Deepali Mathur, Bikash Kumar Mishra, Soumyashree Rout, Francisco Jose Lopez-Iranzo, Gerardo Lopez-Rodas, Jayalakshmi Vallamkondu, Ramesh Kandimalla, Bonaventura Casanova

**Affiliations:** 1 Department of Neurology, Apollo Hospitals, Bhubaneswar 751005, India; matdeepali@gmail.com (D.M.); mbikashkumar@hotmail.com (B.K.M.); rout.soumyashree85@gmail.com (S.R.); 2 Hospital of Requena, Generalitat Valenciana, 46340 Requena, Spain; fj.lopeziranzo92@gmail.com; 3 Department of Biochemistry and Molecular Biology, Institute for Health Research INCLIVA, University of Valencia, 46010 Valencia, Spain; 4 National Institute of Technology, Warangal 506004, India; vlakshmij@gmail.com; 5 Department of Biochemistry, Kakatiya Medical College, Warangal 506007, India; ramesh.kandimalla@gmail.com; 6 Department of Applied Biology, Indian Institute of Chemical Technology (CSIR-IICT), Hyderabad 500007, India; 7 Unitat de Neuroimmunologia, Hospital Universitari i Politècnic La Fe, la Universitat de València, 46026 Valencia, Spain

**Keywords:** biomarkers, predictive, diagnostic, prognosis, treatment response monitoring, multiple sclerosis

## Abstract

Multiple sclerosis (MS) is a complex disease of the central nervous system (CNS) that involves an intricate and aberrant interaction of immune cells leading to inflammation, demyelination, and neurodegeneration. Due to the heterogeneity of clinical subtypes, their diagnosis becomes challenging and the best treatment cannot be easily provided to patients. Biomarkers have been used to simplify the diagnosis and prognosis of MS, as well as to evaluate the results of clinical treatments. In recent years, research on biomarkers has advanced rapidly due to their ability to be easily and promptly measured, their specificity, and their reproducibility. Biomarkers are classified into several categories depending on whether they address personal or predictive susceptibility, diagnosis, prognosis, disease activity, or response to treatment in different clinical courses of MS. The identified members indicate a variety of pathological processes of MS, such as neuroaxonal damage, gliosis, demyelination, progression of disability, and remyelination, among others. The present review analyzes biomarkers in cerebrospinal fluid (CSF) and blood serum, the most promising imaging biomarkers used in clinical practice. Furthermore, it aims to shed light on the criteria and challenges that a biomarker must face to be considered as a standard in daily clinical practice.

## 1. Introduction

Multiple sclerosis (MS) is a chronic, inflammatory, and autoimmune disease of the central nervous system (CNS) that causes damage to myelin sheaths and axons. It affects more than two million people worldwide and 5–20 per 100,000 persons in India [1,2]. Young individuals aged between 20 and 40 years are mainly affected, although MS also develops in children, teenagers, and the elderly [3]. According to Lubin et al.’s classification, MS is categorized into relapsing and progressive stages, with disease activity and/or progression [4]. Ninety percent of patients are affected with the relapsing remitting clinical form of MS (RRMS). The majority of these patients eventually transition into secondary progressive MS (SPMS), which causes further deterioration and neurological disability [5]. The progressive form of MS is comprised of primary progressive MS (PPMS) and SPMS. Approximately 15% of patients are affected with PPMS, which is considered the subtype with the worst prognosis [6]. Due to heterogeneous clinical presentation of MS, there is no specific ‘diagnostic test’ available in the laboratory, leading to delays in diagnosis and determining a prognosis. Therefore, accurate diagnostic tools are required to understand the development and progression of disease, and the use of blood and cerebrospinal fluid (CSF) biomarkers would aid in the diagnosis, prognosis, and monitoring responses to current treatments [7]. The later objective would be of particular interest in SPMS, since most of the patients that have this MS-subtype do not respond well to new disease-modifying therapies (DMT) used to treat MS [8,9].

Neuromyelitis optica spectrum disorder (NMOSD) is historically considered to be the Asian form of the optic-spinal form of MS. Initially, NMO was considered as an isoform of MS and not as a distinct entity of autoimmune and demyelinating CNS disease. However, the discovery of NMO-specific IgG antibodies found in the serum of NMO patients, mainly against aquaporin 4 protein and expressed in astrocytes, allowed the differentiation of MS from NMO patients. Moreover, extensive clinic research and the development of highly sensitive and specific methods to assess myelin oligodendrocyte glycoprotein antibody (MOG-Ab) disease allowed the diagnosis of a group of patients with MOG antibodies, which are also clinically and phenotypically different from MS or NMO. Therefore, MOG-Ab disease is now viewed as a separate entity that requires a specific, differentiated treatment. MOG-Ab disease is also detected in patients with acquired demyelinating syndrome [10,11,12], clinically isolated syndrome, optic neuritis, transverse myelitis, NMO spectrum disorders, and MS [13,14].

Therefore, the aforementioned data clearly justify a detailed investigation of biomarkers to better understand the factors that contribute to the development of MS, its exacerbation into the various subtypes, the effects of treatment, and the disease prognosis. Biomarkers would also be useful in differential diagnoses between MS and other CNS demyelinating diseases with similar signs or symptoms.

In recent years, several remarkable reviews have been published that highlight the importance of biomarkers in the prediction, diagnosis, and outcome of treatments in MS e.g., [15,16,17,18]. In this physically and mentally disabling disease, being able to make relevant information on the potential best biomarkers easily accessible to MS-clinicians is essential for daily practice. Bearing this in mind, the objective of this review is to describe and analyze the main biomarkers clinically available, along with their present and potential future uses in MS patients.

## 2. The Criterion to Be Considered as a Standard Biomarker

The characteristics used to classify a biomarker as ideal are as follows:

(1) It must have the ability to differentiate between a patient and a healthy individual;
(2) It must be expressed at an early stage before the disease progresses;
(3) It must be easy to evaluate, safe for patients, and informative for subsequent management of the disease;
(4) It must offer reproducible results. Furthermore, the biomarker must be sensitive, specific, and must have a clear predictive value.

In this way, the identification of specific biomarkers can help to diagnose the disease, understand the progression of the disease, and what the response to therapeutic regimens would be.

In MS, body fluids such as blood and CSF have different merits and demerits for use in identifying the specific biomarkers they contain. The detection of biomarkers in a blood sample is a safe, fast, and simple technique, and, contrasting CSF, biomarkers can be measured at different time points without undue inconvenience to patients. However, there are certain demerits to the blood sample method. For instance:

(1) The measurement of biomarkers in a blood sample may not necessarily reflect changes in the CNS, unlike CSF;
(2) In blood samples, markers are affected by many clinical and biological processes;
(3) The concentration of biomarkers in blood may be lower than the corresponding amount in the CSF.

Although measuring a biomarker in the CSF directly has the advantage of reflecting changes in the CNS, these samples must be obtained by lumbar puncture, which is a difficult and traumatic process that cannot be repeated with the frequency of a simple blood sample.

## 3. Challenges in the Development of Biomarkers

There is no doubt that there are several obstacles that must be overcome in order to establish biomarkers as ideal for clinical use. For instance, there are different detection strategies and kits available on the market to detect molecular biomarkers. However, even small variations in the available detection methods can result in significant differences for the same tested biomarker, and thus seriously influence the significance value of the biomarker. For example, for interleukin IL-21, two different ELISA kits were used to test its potential for possible use as a biomarker, and they found significantly different results [19,20]. It is therefore essential to employ different detection methods to confirm the potential validity of a biomarker.

Likewise, the results can vary significantly when the validity of a biomarker is established in a small population and subsequently applied in a large independent cohort. For instance, studies with a small number of patients showed that anti-myelin oligodendrocyte glycoprotein (MOG), anti-myelin basic protein (MBP), or anti-KIR4.1 antibodies could serve as a predictor of the development of MS after a first demyelinating event [21,22]. However, subsequent studies with large population cohorts could not confirm this prediction [23,24]. Although there are other challenges that potential new biomarkers face, the robustness of the assay used and its application in large populations must be taken into account when developing them, in order to minimize the error of their use in patients.

## 4. Classification of Biomarkers

Biomarkers are classified according to:

(A) Personal susceptibility or predictiveness;
(B) Diagnosis;
(C) Prognosis;
(D) Disease-associated activity;
(E) Response to treatment in different disease courses of MS (Table 1).

In this review, we focus primarily on the first four criteria, given that responses to clinical treatments frequently use biomarkers from these other groups, some examples of which will be discussed throughout this review.

### 4.1. Predictive Biomarkers

The predictive biomarkers are useful for identifying particular individuals who are at risk for developing MS. People who are at an increased risk of developing MS and would benefit from targeted screening include:

(A) Children and siblings of MS patients;
(B) Healthy individuals diagnosed with clinically isolated syndrome (CIS) and other disorders related to the nervous system.

These risk groups were determined by identifying risk factors that correlate with MS [25]. The immune reaction to the Epstein–Barr virus (EBV) or human herpes virus (HHV)-6, or the presence of anti-myelin oligodendrocyte glycoprotein and anti-myelin basic protein (MBP) antibodies are good examples of predictive biomarkers (Table 2).

- Epstein–Barr virus (EBV)

There is considerable experimental evidence suggesting an association between EBV and MS due to the similarity in the geographical distribution of the appearance of both diseases, the existence of a greater number of reported cases of infectious mononucleosis in MS patients, and higher titers of specific EBV-associated antibodies in patients with an increased risk of MS [26]. Abrahamyan et al. studied the prevalence of Epstein–Barr nuclear antigen (EBNA)-1 and viral capsid antigen (VCA) antibodies in serum from a cohort of 901 patients with CIS or early RRMS [27]. The results showed that 100% of the patients with CIS/RRMS were EBV-seropositive, thus suggesting that a negative EBV serology in patients with suspected inflammatory disease of the CNS should alert clinicians to consider diagnoses other than MS. In addition, in the largest population of EBV-CIS evaluated to date (a cohort of 1047 cases of CIS), only one was seronegative for EBNA-1, demonstrating that, while it is possible to be truly seronegative for EBV and develop MS, it is extremely rare [28].

- Human herpesvirus type-6 (HHV-6)

The experimental results show high levels of the expression of the HHV-6 virus in oligodendrocytes close to the MS plaques [65], suggesting that there is a definitive causal relationship between HHV-6 infection and the development of MS [66]. Evidence of HHV-6 neurotropism was also found in MS from viral DNA found in the brain and CSF of MS patients. Furthermore, in clinical case-control studies, increased expression of HHV-6 genes, with higher levels of virus mRNA and proteins, were also found in the oligodendrocytes of MS patients [67,68,69]. In a more recent work, Dominguez-Mozo et al. described that higher titer of IgG HHV-6 antibodies are related to the occurrence of disease relapses in patients with MS and that treatment with natalizumab drastically reduces both the relapse rate and IgG HHV-6 antibody titers. Based on these data, the authors highlight the potential role of these antibodies not only as predictive factors, but also as early biomarkers of drug response in patients with MS [70].

- Anti-MOG and anti-MBP antibodies

Oligodendrocytes are the myelin-forming cells of the CNS, and therefore any cellular damage to oligodendrocytes, for instance by autoantibodies, can lead to the loss of myelin sheath structure. The presence of antibodies against myelin proteins in the serum reflects an autoimmune attack against CNS myelin. Thus, anti-myelin autoantibodies, such as MOG and MBP, in the serum of patients with CIS can be considered as predictive biomarkers of disease. Berger et al. found antibodies against MBP in the serum of CIS patients who developed MS [21], and pointed out that the presence of anti-MBP antibodies in childhood increases the risk of demyelinating encephalomyelitis [112]. Subsequent studies showed that MS patients with anti-MOG antibodies are at an increased risk of developing MS and have a higher relapse rate [113]. Despite these results, the studies are not entirely conclusive since, for instance, Kuhle et al. observed that a clear connection between anti-MOG and anti-MBP presence and CIS to MS conversion is not apparent. For instance, Kuhle et al. observed that a clear connection between anti-MOG and anti-MBP presence and CIS to MS conversion is not apparent [114]. Furthermore, Kuerten et al. used microarrays to analyze antibodies against 205 myelin antigens in a cohort of 13 MS patients. Microarray significance analysis identified a subset of 64 myelin antigens, including MOG and MBP, for which widely elevated levels of anti-myelin autoantibodies could be detected in the plasma of MS patients. Despite this, the authors noted that the levels of significance were not high enough to serve these antibodies as a good predictive clinical biomarker for MS [114]. Based on these results, and those obtained by other authors (reviewed in [115]), anti-MOG antibodies would not be adequate biomarkers for the diagnosis or prognosis of MS, but rather for its differential diagnosis with MOG^+^-CNS demyelinating disease representing a new distinct disease entity.

### 4.2. Diagnostic Biomarkers

The purpose of diagnostic biomarkers is to confirm that a patient has a certain pathology, such as MS, and thus allow clinicians to discriminate between them and healthy people or those suffering from another potentially related disorder (Table 2 and Table 3). For MS, the diagnosis of the first clinical events of relapsing-remitting MS with disseminated inflammation in space can be confirmed by magnetic resonance imaging and by the existence of oligoclonal immunoglobulin bands detected in CSF. This modified McDonald MS diagnostic criteria include oligoclonal bands replacing the classic criteria of diffusion over time. Thus, when published MS data were compared by applying classic 2010 MS diagnostic criteria or 2017 modified McDonald MS diagnostic criteria, it was found that around 37% of the patients were correctly diagnosed with the 2010 criteria, and surprisingly, the number increased to 68% when the 2017 criteria were applied [174]. Therefore, the recent criteria, which include the existence of detected oligoclonal bands in CSF, provides a faster and more cost-effective approach to the diagnosis of MS than the classical criteria. However, the McDonald’s 2017 criteria have limitations when they are applied to patients with an atypical clinical syndrome or other inflammatory disorders of the CNS. These patients require expert MS clinicians for an accurate diagnosis of the disease.

#### 4.2.1. MRI as Diagnostic Biomarker

Magnetic resonance imaging (MRI) is probably the single most important tool for diagnosing MS. This technique makes it possible to determine localized lesions in SNC. In MS, if lesions are found in the white matter of the brain, this indicates that MS is developing from CIS. The MRI study for MS includes:

(A) T1-weighted or longitudinal magnetization relaxation time;
(B) T2-weighted or transverse magnetization relaxation time;
(C) A post-contrast scan. T1-weighted lesions are used primarily to detect any abnormalities in the integrity blood-brain barrier (BBB). Hypointense T1 lesions (also referred to as black holes) are used as a marker representing the loss of axons that occur during the development of MS.

**Figure 1:**
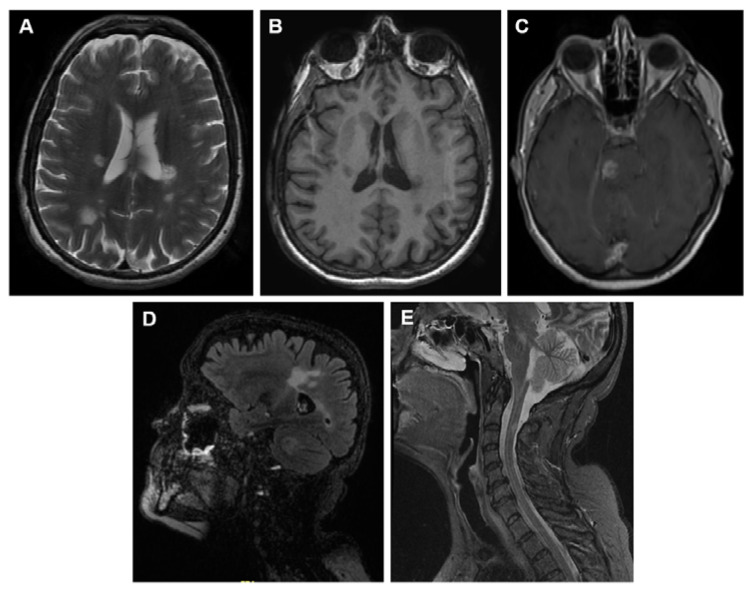
Representative images of MS lesions in the CNS obtained by the magnetic resonance techniques (MRI) most used for diagnosis in daily clinical practice. (**A**) Brain MRI T2-weighted. (**B**) Brain MRI T1-weighted. (**C**) Brain MRI T1-weighted after gadolinium administration. (**D**) Brain MRI FLAIR-weighted. (**E**) Spinal cord MRI STIR sequence.

MR imaging is classified into conventional and unconventional techniques (Table 3) and Figure 1 shows clinical examples of these techniques used in MS patients.

##### Conventional Image Techniques

- T2- weighted MR imaging

MS lesions are typically detected on T2-weighted MRI images. T2-weighted hyperintense lesions have considerable diagnostic importance as they involve multiple clinical mechanisms of MS, such as axon loss, demyelination, and inflammation and oedema in the CNS. Through this method, both white matter (WM) and gray matter (GM) lesions can be identified. The WM and GM lesions indicate whether there is focal and cortical demyelination, and also whether there is significant axonal loss. Therefore, it is undoubtedly the most important imaging biomarker for studying neurodegeneration during MS [29].

- T1-weighted images with gadolinium enhancement

T1-weighted lesions are used to detect BBB dysfunction in MS. T1-weighted images also provide data on CNS neurodegeneration by analyzing black holes and atrophy. Black holes describe axonal damage and destruction of neuronal tissue, while atrophy shows axonal loss that would likely occur through tissue damage within the lesions. Quantifying the number and measuring the volume of T1 black holes would correlate well with MS disability, which is why it has been proposed in the literature as a potential biomarker for CNS neurodegeneration [30,31]. MRI with Gd enhancement is useful for analyzing active inflammation, since Gd would easily enter the CNS through BBB damage [32,33].

- Fluid Attenuated Inversion Recovery Sequence (FLAIR)

Due to the presence of excessive water and a high spin density in MS lesions, sometimes it is not possible to get a better figure of parenchymal changes in MS development. To avoid these difficulties, the FLAIR sequence technique suppresses the resonant signals of water, enhancing the hyperintense signals of the MS lesions. In this approach, intraparenchymal changes can be detected in MS, especially in the cortical and juxtacortical areas [34,35]. The technique is not sensitive enough to detect lesions in the periventricular region, which are usually detected using the T2-FLAIR sequence [34].

- Proton density (PD)-weighted spin-echo (SE) images

Long repetition time (TR) and shorter echo form PD-weighted (proton density weighted) image forms are also well-known image biomarkers. Eco sequences were used earlier in the form of a fast spin-echo (FSE). Dual and multi-echo sequences can also be used to get PD and T2 weighted images. According to Chong in 2016, PD-FSE exposes larger lesions, which appears as numerous smaller lesions in T2- FSE images. Additionally, 32% more cervical cord lesions are identified through this procedure than through T2- FSE images, thus providing more information about neurodegeneration [36].

Images with a long repetition time (TR), shorter echo PD-weighted (proton density weighted) or, previously, fast spin echo (FSE) images are used as a useful technique in MS clinic. To obtain PD- and T2-weighted images, the more sensitive dual and multi-echo sequences can also be used. As highlighted by Chong et al., PD-FSE exposes larger lesions found in the form of numerous smaller lesions on T2-FSE images. By this procedure, 32% more extra cervical cord lesions are identified than with T2-FSE imaging, although the latter provides more information on neurodegeneration in the CNS [36].

##### Non-Conventional Image Techniques

- The Magnetization Transfer Imaging (MTI)

MTI is a type of MRI technique that is applied to expose the interaction between protons that are present in three states:

(a) Bound to macromolecules;
(b) In free water;
(c) As water in the hydration layer between macromolecules and free water.

MTI detects and differentiates the specific lesions that define MS to determine the severity of the disease and to describe the pathological changes that occur during MS development [35,37].

MTR is a sensitive method by which the pathogenesis of MS of myelinated white matter lesions can be assessed relative to the intact white matter, such that the decrease in the MTR value is correlated with both demyelination and axonal loss [38,39]. MTR can also provide information on the degree of optic nerve demyelination during optic neuritis. The MTR value of the optic nerve decreases, which is correlated with the thickness of the retinal nerve fiber layer (RNFL) and axonal loss, making it relevant for evaluating pathological and paraclinical visual changes during the disease [40].

- Diffusion-Weighted (DWI) and Diffusion Tensor (DTI) Images

DWI is another MRI technique for evaluating Brownian motion of H_2_O. This technique studies the anatomy and potential pathologies affecting white matter (NAWM). The DWI estimates the so-called apparent diffusion coefficient (ADC) of water in the brain, which increases if there are alterations in cell structure and white matter tracts [41]. The technique is capable of distinguishing between different pathologies that affect the CNS, such as ischemia, infection, tumors, neurodegeneration, etc., by calculating the microscopic movement of water molecules whose diffusion is atypical in EM plaques [42]. Although this technique is useful for analyzing ischemic cerebrovascular accidents, its status as a marker of MS is not well established and it is used less than other MRI techniques. For instance, DWI is unable to measure the volume of tissue loss in MS lesions and cannot reveal modifications generated by other pathologies that occurred in white matter.

DTI is an MRI refinement method that evaluates the three-dimensional diffusion of water in multiple directions in space. DTI provides much more information on the pathogenesis of MS compared to T1- or T2-weighted MRI techniques. In DTI, several physical parameters are evaluated, such as:

(a) Axial diffusivity (DA)
(b) Radial diffusivity (RD);
(c) Mean diffusivity (MD);
(d) Fractional anisotropy (AF) [42].

The DA assesses the loss of axons while the RD is associated with the status of the myelin layer. In the early stages of MS, the DA value is low in normal-appearing white matter (NAWM), increasing in the later stages of MS [43]. On the other hand, DTI uses the failure analysis (FA) process to reveal the global direction of water diffusion, which increases in white matter tracts and decreases in fibers disorganized by MS [44].

The evaluated DTI values serve as biomarkers for the diagnosis and evolution of MS. MD has been established as a useful and predictive biomarker for estimating the onset or relapse of MS, since it is sensitive to changes in the tissues that precede the breakdown of the blood–brain barrier (BBB) for at least 5 months and can predict injury after restoration of the BBB [45]. DA is used as a potential biomarker of axonal degeneration and RD is used to determine the degree of demyelination over the course of MS [46,47,48].

- Magnetic Resonance Spectroscopy (MRS)

Nuclear magnetic resonance spectroscopy (MRS) is a non-invasive technique that estimates the cellular metabolism in the CNS. The MRS measures the biochemical levels of molecules such as *N*-acetylaspartate, choline, creatine, glutamate, and glutamine in various pathologies, along with their deviation from natural physiological levels, as occurs in the pathogenesis of MS. Specifically, a decrease in the level of *N*-acetylaspartate is considered a marker of neuronal/axonal loss, while choline estimates the increase in rotational cell membrane components that is observed during demyelination or gliosis. For its part, glutamate is used as a biomarker of acute inflammation, a reduced level of *N*-methyl aspartate indicates a decrease in oedema in MS lesions, and GABA is decreased in the SPMS form of MS [49,50].

- Optical Coherence Tomography (OCT)

Optical coherence tomography (OCT) is used to take images of axons and the unmyelinated axonal layers of the retina (RNFL). The variation of the RNFL reflects neurodegeneration and oedema in MS patients. Lower values of RNFL are considered a high-resolution, objective, non-invasive, and easily quantifiable in vivo biomarker of MS and represent the axon loss associated with cerebral atrophy [51,52].

- Positron Emission Tomography (PET)

Positron emission tomography (PET) is an imaging technique used to estimate cell and tissue metabolism through the in vivo incorporation of a radiopharmaceutical. The heterogeneity of MS lesions, and the changes in inflammation of the NAWM and GM in the course of MS, can be obtained through PET images. The focal point of this technique is to produce functional images of the brain’s innate immune system, that is, of the microglia and macrophages. For instance, activated microglia secrete oxygen radical species (ROS) and pro-inflammatory cytokines in response to the neural injury that causes the inflammation characteristic of MS. Therefore, the PET technique is used as a biomarker and shows the process of neurodegeneration and neuroinflammation in the CNS of patients with MS [53,54].

#### 4.2.2. Diagnostic Biomarkers in CSF and Serum

- Oligoclonal bands (IgG) and (IgM)

Oligoclonal bands (OCB) are immunoglobulins (Ig) produced intrathecally and, although they are considered an immunological characteristic of MS, they are not exclusive to MS, as they are also found in other CNS pathologies [71,72,73]. OCBs are present in the CSF of more than 95% of MS patients, are absent in serum, and serve as an important criterion for the diagnosis of MS (Table 3). Among the oligoclonal bands, those of the IgG type (OCGB), produced by B cells, are possibly the most important biomarker attributed to CNS demyelinating spectrum disorders. The OCGB serves as a diagnostic element in patients with Clinically Isolated Syndrome (CIS), and is also associated with the development of Clinically Defined MS (CDMS), due to the presence of a higher level of IgG in the CSF of these patients. The presence of OCGB is also essential to predict the progression of Radiologically Isolated Syndrome (RIS) patients to CIS and from CIS to MS [74,75], whose sensitivity and specificity is 88% and 86%, respectively [76]. The presence of high levels of OCGB in the CSF of patients with CIS favors the transition to CDMS [77,78] and the subsequent progression of patients to definitive MS [79].

The presence of OCGB is also useful in predicting Optic Neuritis (ON) in MS [80,81], although they are not able to report the intensity of a second relapse. Several lines of evidence indicate that patients with OCGB present in their CSF exhibit a higher level of inflammatory activity, which leads to significant tissue damage [82,83,84], a greater degree of lesions, and greater brain atrophy [85,86,87,88].

- Immunoglobulin G index (IgG index)

The levels of IgG and IgM OCBs released intrathecally in MS patients indicate the clonal extension of B cells and plasma cells in the CNS. The relative amount of IgG in the CSF, compared to that present in serum, is evaluated by the IgG index. The IgG index is estimated as the ratio of IgG to albumin in CSF compared to the ratio of IgG to albumin in serum [89]. On the other hand, the albumin quotient (albumin in CSF/albumin in serum) indicates the alteration to the integrity of the blood–brain barrier in MS [90]. It has been estimated that a patient may be diagnosed as having MS when the IgG index exceeds the value of 0.7, which means an increased synthesis of intrathecal IgG antibodies in the CNS which in turns triggers the symptoms of MS. Thus, the IgG index serves as an important biomarker for the diagnosis of MS and is routinely determined during the MS diagnostic.

- Kappa Free (KFLC) and Lambda Free Light Chains (LFLC)

Plasma cells produce KFLC and LFLC during antibody synthesis, and these proteins can be detected in both serum and CSF of patients [91]. Presslauer et al. found that MS patients have a higher amount of KFLC secreted in their CSF and in their blood serum [92]. Rinker et al. demonstrated a clear correlation between the presence of an increasing amount of KFLC and the occurrence of future disabilities in MS patients [93]. Moreover, Villar et al. found that KFLC levels increase in the CSF of CIS patients and that these altered levels lead to the conversion of CIS to CDMS, suggesting its use as a marker of MS progression [94]. It has also been proposed that the LFLC subunit can be used as a predictive biomarker for intrathecally produced immunoglobulins in inflammatory disorders of the CNS [116].

- Anti-Aquaporin-4 (AQP4) antibodies

Aquaporin-4 (AQP4) is expressed in CNS astrocytes and its function is to transport water through the cell membrane and maintain the homeostatic balance within the CNS. MS patients lack the expression of this protein, while 38–75% of patients with Neuromyelitis Optica (NMO) have AQP4 antibodies [117]. Since NMO is a rare disease in which the immune system attacks myelin surrounding the optic nerve and spinal cord, the differentiation between NMO and MS is challenging due to their similar clinical features. Patients with NMO have specific IgG antibodies in their serum directed against AQP4, which is expressed in astrocytes. Therefore, the specific immunoreactivity of this biomarker helps to differentiate between patients with NMO and MS, and also improves the determination of other disorders related to immunity also affecting the CNS [154,155].

### 4.3. Biomarkers of Prognosis

Prognostic biomarkers are used to predict the potential response of a patient to treatment in terms of efficacy and/or safety, which allows clinicians to make the most appropriate clinical and therapeutic decisions.

- Oligoclonal bands (IgM)

The IgM-type immunoglobulins (OCMBs) were shown to correlate with MS activity [156] and predict an aggressive course in patients with RRMS during the early stages of the disease [157,158,171,172]. The presence of OCMB is associated with an increase in retinal axonal loss in MS [171], the thinning of the retinal nerve fibre layer [172], and its deposition influences inflammatory processes in the brain, generating greater lesions in the CNS [173].

- Chitinase-3-Like-1 precursor (CHI3L1)

Several lines of evidence indicate that the levels of the extracellular glycoprotein CHI3L1 are found to be elevated in CSF of patients with inflammatory diseases of the CNS. This biomarker is expressed in astroglia, in WM plaques, in NAWM, and in the brain lesions of MS patients [55]. Canto et al. found elevated CSF CHI3L1 levels that correlate with the rapid development of disability during MS progression. CHI3L1 can be used as a biomarker in the transformation of CIS into CDMS [56], being elevated in patients with RRMS and SPMS compared to healthy individuals [57]. Likewise, higher levels of CSF CHI3L1 are found in T1 and T2 lesions and in the brain parenchymal fraction of MS patients. Therefore, it is suggested that CHI3L1 could be another promising prognostic biomarker of MS.

- Neurofilaments (NF)

Neurofilaments (NFs) belong to type IV intermediate filaments and shape neurons. In the CNS they are abundant in the cytoplasm of neurons and are composed of four subfilaments of different molecular weights:

(a) NF-L (polypeptide light chain) of 68 kDa;
(b) NF-M (neurofilament of medium size) of 150 kDa;
(c) NF-H (heavy chain) from 190 to 210 kDa and α-internexin [95].

If axonal damage occurs in the CNS, the filaments are secreted into CSF or blood serum [96]. The NF-L light chain can be considered as a prognostic biomarker that provides information on the transformation of CIS into RRMS [97,98]. Likewise, a high level of NF-L in CSF is considered a predictive marker of disease severity and progression to SPMS [99], and of disability and cognitive impairment during the conversion of patients to CDMS [100,101]. On the other hand, Gunnaarsson et al. observed that NF-L is associated with CNS damage, so it can also be used as a neurodegeneration biomarker in MS [102]. Kuhle et al. further demonstrated that NF-L is a marker of tissue damage and disease activity in patients with RRMS, suggesting that it is also a prognostic biomarker [103]. Patients with RRMS and SPMS have a high concentration of NF-H, and this NF is used as a prognostic marker in the development of MS and the future disability of patients [104,105].

- miRNAs

Non-coding micro RNA molecules (miRNAs) can be detected in both serum and CSF samples. Although mRNAs are highly unstable, the miRNAs are relatively highly stable in biological fluids. Many studies of miRNA have revealed that alteration in the level of some miRNAs is related to the conversion of CIS to MS [118]. For instance, a higher level of miRNA-922 is found in patients with CIS and is associated with increased transformation of CIS into RRMS [119]. In another study, Bergmann et al. found that miRNA-150 present in plasma can function as a prognostic marker, since it influences in the conversion of CIS to MS [120]. Furthermore, Ma et al. showed that miR-19a, miR-21, miR-22, miR-142-3p, miR-146a, miR-146b, miR-155, miR-210, and miR-326 are up-regulated in MS [121]. In contrast, miR-15a, miR-15b, miR-181c, and miR-328 are downregulated in MS patients [121]. Therefore, miRNAs are being considered as promising prognostic biomarkers in patients with MS, as they are able to adequately predict the conversion of patients with CIS to MS.

### 4.4. Biomarkers of Dysfunction and Pathology

MS-type neurological disease activity is defined as the appearance of new neurological symptoms, the recurrence of a previous condition, identifiable radiological activity, or the progression of disability. Clinicians assign a score that predicts whether symptoms have resumed or ceased and whether it is necessary to continue or modify medication to control the disease.

#### 4.4.1. Biomarkers of Immunological Dysfunction

- Cytokines

Neuroinflammation occurs during the relapsing phase of MS, releasing numerous cytokines and chemokines into the CSF, which in turn produces CNS lesions characteristic of MS. Many articles have been published in the literature that describes the changes in certain cytokines during the course of MS, and that are detectable in the CSF of patients [58,59,60,61,62], or in their serum [62,63,64,65]. Various authors have proposed cytokines as potential biomarkers of immunological dysfunction in MS.

The chemokine CXCL13, interacts with the CXCR5 receptor, and results in the activation of B and T helper cells in demyelination lesions. Furthermore, a higher level of CXCL13 is found to be associated with the conversion of CIS to MS [58]. Mouzaki et al. found that, through cytokines, it is easy to differentiate MS patients from other inflammatory CNS disorders [59]. Kim et al. also showed that any imbalance in the IL-1 signaling leads to CNS demyelination [60]. Huang et al. investigated protein biomarkers in CSF and plasma using a highly sensitive proteomic immunoassay. The cases from two independent cohorts were compared with healthy controls and patients with other neurological diseases. Ten up-regulated proteins were identified in CSF, including CCL11, IL-12B, CD5, MIP-1a, and CXCL9 in MS patients [61]. Moreover, CCL11 was associated with disease duration, particularly in patients with SPMS subtype [61]. Bai et al., in a meta-analysis of 226 studies with 13,526 MS patients and 8428 healthy controls, showed that 13 CSF cytokines are significantly associated with MS, and among them, CCL21, IL-15, CCL19, CCL11, CCL3, and CXCL13 showed larger standardized mean differences (measured as ES parameter or effective sizes differences between groups with statistical significance) in cytokine concentrations between MS patients and healthy controls [62].

Cytokines have also been proposed as potential biomarkers in blood serum, a clearing fluid that is easier and less traumatic to obtain, which is why it would be highly useful in daily clinical practice. For instance, the relapse rate and prevalence of MS patients was correlated with the levels of interleukin IL-6 in their blood serum [63] and any imbalance in IL-1 signaling leads to increased CNS demyelination [64]. Mouzaki et al. pointed out that measuring some cytokines in serum may allow clinicians to differentiate MS patients from other CNS inflammatory disorders at an early stage [59]. In the same meta-analysis by Bai et al., of the 37 serum cytokines analyzed in the meta-analysis, 21 cytokines were significantly associated with MS. Among these, CCL20, IL-23, IL-21, IL-12p40, IL17F, IL22, and IL2R had large ES values which differentiated between MS patients and healthy controls [62].

#### 4.4.2. Biomarkers of Demyelination

- Myelin basic protein (MBP)

Although the levels of the MBP protein increase in the CSF of patients with MS during acute demyelination, they are not considered a good prognostic biomarker [106,107]. This is because MBPs in CSF tend to remyelinate demyelinating lesions, but the reverse pathway has not been yet demonstrated [108,109]. The literature reveals that a higher level of MBP has been found in the CSF of MS patients [110] and that MBP levels increase dramatically during the relapse of MS patients [111]. Despite this, it is not considered a good pathology biomarker, given the inconsistency of the results obtained.

#### 4.4.3. Biomarkers of Axonal Damage

- Neurofilaments (NF)

MS patients with high levels of axonal degeneration and neuronal death have a higher level of neurofilament type NF-H in the progressive clinical course of MS [122,123]. Likewise, higher levels of NF-H have been found in patients with CIS and RRMS [124], and correlate with the relapse activity of patients with CIS and RRMS [125]. For its part, the NF-L type, after dissociation, disperses from the parenchyma into the CSF due to its low molecular mass and hypophosphorylation [125], and a high level is also found in patients with MS or CIS [126,127]. It has even been shown that NF-L levels increase in the CSF during acute MS relapse and in MS patients with higher relapse rates. Finally, it was reported that in cases of transition from RRMS to SPMS, a higher level of NF-L is also found [97]. Therefore, neurofilaments are good candidates to be used as a prognostic biomarker to determine axonal damage and estimate the efficacy of MS treatment.

- Microtubule-associated protein Tau

Tau protein can be used as a biomarker of axonal loss in patients with MS [128]. When the abnormal phosphorylation of the protein occurs, the microtubules are unstable, generating insoluble tau that acts as a neurotoxic agent. Neurotoxic tau results in the development of common neurodegenerative diseases such as MS [129,130]. Following neuronal injury, tau protein is released into serum and CSF [131], abnormally hyperphosphorylated and in an insoluble tau form, can be found in patients with SPMS and PPMS, leading to the progression of the disease [132,133]. Patients with CIS show elevated levels of both tau and NF-L, which favors the transformation of CIS into CDMS, and the progression of MS [124,133].

- *N*-acetylaspartate (NAA)

Studies have revealed that reduced levels of NAA are found in MS lesions, surrounding NAWM and cortical grey matter [134]. Disease progression in MS and disability are associated with a low level of NAA [128]. Other studies have shown that a decreased level of NAA is present in CIS patient’s grey matter and in some white matter lesions [135,136]. Narayanan et al. found that MS patients who are treated with interferon-β 1b for one year express a higher concentration of NAA [137].

- Amyloid-precursor protein (APP)

Although the accumulation of protein Aβ is seen primarily in Alzheimer’s disease (AD), recent studies have indicated a link between amyloid-β and MS. Increasing evidence suggests that myelin damage results in proteolytic processing of APP. In damaged axons, the cytoskeleton causes disruption of axoplasmic flow, possibly due to a massive influx of calcium across the membrane [138,139]. Changes in axoplasmic flow lead to the deposition of APP in the axons, indicating a failure of axonal transport. Mattson et al. found that the level of α-Sapp and β-Sapp decreased in the CSF of MS patients compared to healthy individuals [140]. APP-positive axons in MS patients are associated with the development of CNS lesions [141]. Therefore, this protein is being used as a potential biomarker to determine the progression of MS. APP is not only found in reactive glial cells during demyelination, but is also expressed during remyelination, with APP being produced by astrocytes during demyelination [142]. The findings of Gehrmann et al. reveal that a higher concentration of APP is detectable in MS patients compared to healthy individuals [141].

- Ionic Imbalance

In myelinated axons, sodium channels are located in the nodes of Ranvier, while in demyelinated axons, they are distributed along the axons in an ATP-dependent process. This increased demand for ATP leads to the failure of Na^+^/K^+^ ATPase, caused by the presence of immune infiltrates in the CSF of MS patients, which leads to an intracellular increase in the ionic gradient of Na^+^. The increased level of intracellular Na+ reverses the activity of the Na^+^/Ca^2+^ exchanger, resulting in the increase in Ca^2+^ in the axoplasm, and finally in axonal damage and mitochondrial dysfunction [143,144]. Our research group has found that patients with RRMS with IgG and IgM antibodies (IgG^+^/IgM^+^) have less energy to repair axonal lesions, due to repression of genes related to ATP production, so they have more aggressive clinical symptoms and greater inflammation compared to IgG^+^/IgM^−^ patients [145]. Oligodendrocyte progenitor cells (OPC) in MS patients with IgG^+^/IgM^+^ RRMS decrease their ability to repair demyelinated regions, due of lack of sufficient energy to repair neuronal damage, which leads to a poor clinical prognosis in contrast to IgG^+^/IgM^−^ RRMS patients [145,146]. Thus, energy failure and toxic sodium accumulation may initiate a vicious cycle, in which the increased intracellular Na^+^ concentrations may provoke reverse action of the Na^+^/Ca^2+^ exchanger and thus cause Ca^2+^ accumulation, leading to a cellular ion imbalance which increases activation of neurodegenerative signaling cascades [147].

It is certainly a challenge to directly quantify energy expenditure or determine the presence of unbalanced ion concentrations in the CNS in vivo through non-invasive techniques. Zhu et al., designed the ^31^P MRS in vivo technique for the detection of intracellular content in the CNS of high-energy phosphate compounds, such as ATP or phosphocreatine, whose concentrations and relation to Pi can be altered under pathological conditions [148]. For their part, various authors have carried out in vivo studies using the sodium magnetic resonance technique (^23^NaMRI) to estimate pathological levels of sodium present in brain lesions of MS, and to determine the course of the disease in patients with the three clinical subtypes of MS (RRMS, SPMS, and PPMS) [149,150,151,152,153]. These studies highlight the importance of these novel MRI techniques in the study of MS and as potential biomarkers of neuroinflammation and degeneration [153].

#### 4.4.4. Biomarkers of Glial Activation/Dysfunction

- Nitric Oxide (NO)

NO is a signaling molecule in many physiological processes and in pathological processes such as MS. NO levels are increased in the CSF and serum of MS patients compared to individuals with non-inflammatory neurological disorders [159,160]. NO acts as a suppressor of mitochondrial activity by inhibiting cytochrome C oxidase, which decreases mitochondrial energy production [161]. NO has extremely toxic effects on neurons and glial cells, enhancing their apoptosis, and also affects the integrity and permeability of the BBB. This enables pro-inflammatory cells to reach the CNS. NO (X) degradation products, present in MS patients, are especially involved in the destruction of mitochondria, causing tissue hypoxia and accentuating damage in MS lesions [162].

- Reactive Oxygen Species (ROS)

ROS are highly reactive oxygen species due to the presence of unpaired valence electrons. ROS alter the myelin sheaths and myelin-producing oligodendrocytes through oxidation mediated by their free radicals. Significant ROS generation was found in MS patients compared to healthy individuals, leading to cellular and tissue oxidative stress in their CNS [163]. ROS production in MS patients favors the generation of superoxide and peroxynitrite, which are harmful to glial and neuronal cells, accentuating the damage to the CNS. Likewise, higher levels of 7-ketocholesterol, a product of the oxidative degradation of lipids, were observed in the CSF of patients with MS, which in turn is capable of inducing neuronal damage through the activation and migration of microglial cells in CNS [164].

- Glial fibrillary acidic protein (GFAP)

It has been observed that the expression of the intermediate filament protein of mature astrocytes GFAP increases in MS plaques, which would be expected to relate to a high level of astrocyte damage [165,166]. A higher level of GFAP was measured in CSF of patients with SPMS than in patients with RRMS, indicating greater neurological dysfunction in this type of MS, as well as a greater progression of disability [167]. Furthermore, MS patients with greater disabilities have higher GFAP levels compared to patients with lesser disabilities [124]. Even higher levels of GFAP are found during the MS relapse itself, and these levels are maintained for at least 5 weeks [168]. On the other hand, it has been observed that, in the CSF of patients with relapsed NMO, the level of GFAP is significantly higher than in the CSF of patients with relapsed MS [169,170]. This evidence suggests that GFAP can be used as a biomarker to detect the development of MS, indicating a high rate of astrogliosis, and could be used to differentiate between types of MS.

- S100 calcium-binding protein B (S-100B)

S-100b protein is found in astroglia, a small subset of oligodendroglia, and a certain subgroup of neurons. The main function of this protein is to promote neuronal proliferation, oligodendrocyte differentiation, stimulation of calcium fluxes, maintaining astrocyte morphology, and facilitating the astrocyte and microglial activation that occurs intracellularly. Petzold et al. showed that an increasing amount of S100B levels were found in all MS subgroups which indicate cerebral injury [168].

## 5. Conclusions

The dearth of knowledge about the pathophysiology of MS and the clinical variation in MS subtypes makes it implausible to establish a single biomarker which guarantees the full evaluation of the disease. For a long time, numerous investigations have been conducted to identify potential biomarkers that provide meaningful information related to the development of the disease and the possible response of MS patients to treatments. Although progress has been made in the search for this ‘magical unique biomarker’, MS continues to be extremely unpredictable, with more unanswered questions than absolute certainties, the finding of it seems still a long way off. Therefore, to date, there remains no single reliable biomarker that can provide information on the prognosis of MS, distinguish between the different clinical courses of MS, and also predict responses to a specific form of treatment. Despite the various clinical challenges that a biomarker faces, continued advancement in research has resulted in the identification of a group of biomarkers specifically targeting the early stages of the disease. However, there remains a need for a panel of validated biomarkers that are capable of predicting and monitoring the efficiency of the growing number of treatment strategies available, with the aim of reducing the recurrence of relapses, and stopping the progression and disability of patients with MS.

## Figures and Tables

**Table 1:** Classification and clinical uses of biomarkers.

Biomarkers	Description
Predictive	Risk to Develop MS
Diagnostic	Fast Interpretation of Pathological State of MS
Prognosis	Outcome or Course of MS
Disease-Associated Activity	Demonstration of Current MS Condition as Inflammation, Demyelination, Cognitive Dysfunction, etc.
Response to Treatment	Predict Response to Therapy in MS Patients

**Table 2:** Radiological biomarkers and biomarkers identified in CSF and serum samples and their standard clinical use in MS.

Predictive Biomarkers	Diagnostic Biomarkers	Prognostic Biomarker	Biomarkers of Dysfunction and Pathology
Epstein–Barr Virus (EBV) [26,27,28]	Radiological Biomarkers [29,30,31,32,33,34,35,36,37,38,39,40,41,42,43,44,45,46,47,48,49,50,51,52,53,54]	Chitinase-3-Like-1 (CHI3L1) [55,56,57]	Immunological Dysfunction Cytokines [58,59,60,61,62,63,64]
Human Herpesvirus Type-6 (HHV-6) [65,66,67,68,69,70]	Oligoclonal Bands (IgG) and (IgM) [71,72,73,74,75,76,77,78,79,80,81,82,83,84,85,86,87,88,89,90,91,92,93,94]	Neurofilaments (NF) [95,96,97,98,99,100,101,102,103,104,105]	Demyelination Myelin Basic Protein (MBP) [106,107,108,109,110,111]
Anti-MOG and Anti-MBP Antibodies [112,113,114,115]	Immunoglobulin G Index (IgG Index) [116,117]	miRNAs [118,119,120,121]	Axonal Damage Neurofilaments (NF) [122,123,124,125,126,127] Microtubule-Associated Protein Tau [128,129,130,131,132,133] *N*-acetylaspartate (NAA) [134,135,136,137] Amyloid-precursor protein (APP) [138,139,140,141,142] Ionic Imbalance [143,144,145,146,147,148,149,150,151,152,153]
	Kappa Free (KFLC) and Lambda Free Light Chains (LFLC) [154,155,156,157,158]		Glial activation/dysfunction Nitric Oxide (NO) [159,160,161,162] Reactive Oxygen Species (ROS) [163,164] Glial Fibrillary Acidic Protein (GFAP) [165,166,167,168,169,170] S100 Calcium-Binding Protein B (S-100B) [168]
	Anti-Aquaporin-4 (AQP4) Antibodies [171,172,173]		

**Table 3:** Currently imaging biomarkers used in MS, lesions that are mainly detected, and utility and limitations of each technique.

Imaging Biomarker	Lesion Category	References	Utility in MS	Limitations
T2-Weighted	Periventricular Juxtacortical	[29]	Indicate Demyelination and Inflammation	Variations in the MR Images which is Due to Variable Radiofrequency Response in the Brain. Background Noise Makes Difficult to Separate Lesions from White Matter
T1-Weighted	Gd-Enhancing Lesions and Spinal Cord Lesions	[31,32]	Shows About Axonal Loss and BBB Disruption	Need of Reproducible and Reliable Method to Quantify Brain Atrophy
MTR	Gd-Enhancing Demyelinated Remyelinated and Necrotic Lesions	[38,39]	Indicate Changes in Myelin Composition and Axonal Loss	Measurements are Not Absolute and Results Vary Repeatedly as a Function of the Shape, Bandwidth and Frequency of MS Pulse so More Errors Occur
DWI and DTI	Demyelinating and Periventricular Lesions	[41,42,43]	Demonstrates about Demyelination and Axonal Loss	Lack of Pathology Specificity. Required Variable Samples According to Different Type of Lesions and Different Locations of the Brain
MRS	Periventricular and Cortical Lesions	[49,50]	Assess Measure Biochemicals as NAA, GABA, etc. which Reflect Axonal Damage and Shows Abnormalities in Pathology of MS	
OCT	Optic Nerve Lesions	[51,52]	Used to Measure RNFL Thickness and Macular Volume also Gives Data about Axonal Loss, Neurodegeneration	Movement of Patient Can Diminish the Quality of Image
PET	Periventricular Necrotic Juxtacortical and Gd-enhancing Lesions	[53,54]	Assess Inflammation in Cortex, NAWM and Gives Information about Clinical Severity	Invasive and Expensive Tool Not Available all the Time. During Acquisition, if Patient Moves the Activity Will Blur Over Brain Structures. Resolution Will be Degraded and Interpretation of Results Will be Impossible

MTR: Magnetization Transfer Ratio, DWI: Diffusion-Weighted Imaging, DTI: Diffusion Tensor Imaging, PET: Positron Emission Tomography, OCT: Optical Coherence Tomography, MRS: Magnetic Resonance Spectroscopy; RNFL: retinal nerve fiber layer.

## Abbreviations

APP	Amyloid-Precursor Protein
AQP4	Aquaporin-4 Protein
BBB	Blood-Brain Barrier
CDMS	Clinically Defined MS
CHI3L1	Chitinase-3-Like-1 Precursor
CIS	Clinically Isolated Syndrome
CSF	Cerebro Spinal Fluid
DMT	Disease-Modifying Therapies
DTI	Diffusion Tensor Imaging
DWI	Diffusion Weighted Imaging
EBNA	Epstein-Barr Nuclear Antigen
FLAIR	FLuid Attenuated Inversion Recovery Sequence
KFLC	Kappa Free Light Chain
LFLC	Lambda Free Light Chain
MBP	Myelin Basic Protein
MOG	Myelin Oligodendrocyte Glycoprotein
MS	Multiple Sclerosis
MRI	Magnetic Resonance Imaging
MRS	Magnetic Resonance Spectroscopy
MTI	Magnetization Transfer Imaging
MTR	Magnetization Transfer Ratio
NAA	*N*-Acetyl-Aspartate
NAWM	Normal-Appearing White Matter
NF	Neurofilaments
GFAP	Glial Fibrillary Acidic Protein
NMOSD	Neuromyelitis Optica Spectrum Disorders
OCB	Oligoclonal Bands
OCGB	IgG-Type Oligoclonal Bands
OCMB	IgM-Type Oligoclonal Bands
OCT	Optical Coherence Tomography
PD-SE	Proton Density-Weighted Spin-Echo Imaging
PET	Positron Emission Tomography
PPMS	Primary Progressive MS
RIS	Radiologically Isolated Syndrome
RNFL	Retinal Nerve Fiber Layer
RRMS	Relapsing Remitting MS
SPMS	Secondary Progressive MS
S-100B	S100 Calcium-Binding Protein B
VCA	Viral Capsid Antigen
